# Supplementary material for: Autophagy, Apoptosis, the Unfolded Protein Response, and Lung Function in Idiopathic Pulmonary Fibrosis
Source: Cells. 2021 Jun 30;10(7):1642. doi: 10.3390/cells10071642 (PMC8307368; doi:10.3390/cells10071642)
Supplement: Supplementary file 1 [file cells-10-01642-s001.zip › cells-1207677-supplementary.pdf]

cells

Supplementary Material

Communication

Autophagy, Apoptosis, the Unfolded Protein Response, and Lung Function in Idiopathic Pulmonary Fibrosis

Pawan Sharma [1], Javad Alizadeh [2], Maya Juarez [3], Afshin Samali [4], Andrew J. Halayko [5], Nicholas J. Kenyon [3,6], Saeid Ghavami [2,7,8,9,*], Amir A. Zeki [3,6,*]

1 Center for Translational Medicine, Division of Pulmonary, Allergy and Critical Care Medicine, Jane & Leonard Korman Respiratory Institute, Sidney Kimmel Medical College, Thomas Jefferson University, Philadelphia, PA 19107, USA; pawan.sharma@jefferson.edu
2 Department of Human Anatomy and Cell Science, Max Rady College of Medicine, Rady Faculty of Health Sciences, University of Manitoba, Winnipeg, MB R3E 3P4, Canada; alizadej@myumanitoba.ca
3 Davis Lung Center, School of Medicine; Division of Pulmonary, Critical Care and Sleep Medicine, University of California, Davis, CA 95616, USA; mmjuarez@ucdavis.edu (M.J.); njkenyon@ucdavis.edu (N.J.K.)
4 Apoptosis Research Centre, School of Natural Sciences, National University of Ireland, H91 W2TY Galway, Ireland; afshin.samali@nuigalway.ie
5 Department of Physiology and Pathophysiology, Max Rady College of Medicine, Rady Faculty of Health Sciences, University of Manitoba, Winnipeg, MB R3E 3P4, Canada; andrew.halayko@umanitoba.ca
6 Veterans Affairs Medical Center, Mather, CA 95655, USA
7 Research Institute of Hematology and Oncology, Cancer Care Manitoba, Winnipeg, MB R3E 0V9, Canada
8 Faculty of Medicine, Katowice School of Technology, 40-555 Katowice, Poland
9 Autophagy Research Center, Shiraz University of Medical Sciences, Shiraz 7134845794, Iran
* Correspondence: aazeki@ucdavis.edu (A.A.Z.); saeid.ghavami@umanitoba.ca (S.G.)

Citation: Sharma, P.; Alizadeh, J.; Juarez, M.; Samali, A.; Halayko, A.J.; Kenyon, N.J.; Ghavami, S.; Zeki, A.A. Autophagy, Apoptosis, the Unfolded Protein Response, and Lung Function in Idiopathic Pulmonary Fibrosis. *Cells* **2021**, *10*, 1642. https://doi.org/10.3390/cells10071642

Academic Editor: Mojgan Djavaheri-Mergny and Mohammad Amin Moosavi

Received: 18 April 2021
Accepted: 23 June 2021
Published: 30 June 2021

Publisher's Note: MDPI stays neutral with regard to jurisdictional claims in published maps and institutional affiliations.

Copyright: © 2021 by the authors. Licensee MDPI, Basel, Switzerland. This article is an open access article distributed under the terms and conditions of the Creative Commons Attribution (CC BY) license (http://creativecommons.org/licenses/by/4.0/).

RESULTS

Corroborative Analysis of Cell Stress Markers and Lung Function

In order to determine whether our procedure for assessing correlations between cell stress markers and lung function was valid and consistent, we conducted additional analyses using a subset of the original correlations as shown in **Figures 2 and 3**. The matching for each subject between their lung tissue cell stress marker expression and lung function were randomly re-assigned within subjects, then linear correlation analyses were conducted on this dataset.

As shown in **Figure S1A**, correlations for UPR markers XBP1 and BiP were consistent with that seen in the original analysis in **Figure 2.** XBP1% was negatively correlated with FEV1 and FVC and both were statistically significant ($p<0.1$). BiP% was also negative correlated with FEV1, FVC, and TLC ($p<0.1$). Cleaved caspase-3% was similarly negatively correlated with RV ($p<0.1$). Conversely, LC3β puncta% was positively correlated with DLCO ($p<0.1$) also consistent with the original analysis.

As shown in **Figure S1B**, colocalized markers of UPR and autophagy and lung function correlations were also similar to and consistent with data presented in **Figure 3.** For brevity we only evaluated a subset of those comparisons here. Colocalized XBP1 and LC3β puncta was positively correlated with TLC ($p<0.1$), and colocalized LC3β puncta and XBP1 was also positively correlated with FVC ($p<0.1$). While colocalized BiP and LC3β puncta were positively correlated with DLCO ($p<0.10$), colocalized LC3β puncta and BiP were not significantly correlated with any lung function parameters ($p>0.1$, data not shown), similarly consistent with the original analysis in **Figure 3.**

Lung Histology of Study Subjects

H&E staining of representative lung histology samples for both IPF and non-IPF study subjects is shown in **Figure S2.** In panel A, the IPF lungs show clear evidence of alveolar space fibrosis showing extensive collagen and extracellular matrix deposition. In panel B, the non-IPF lungs show normal-appearing alveolar spaces without evidence of fibrosis.

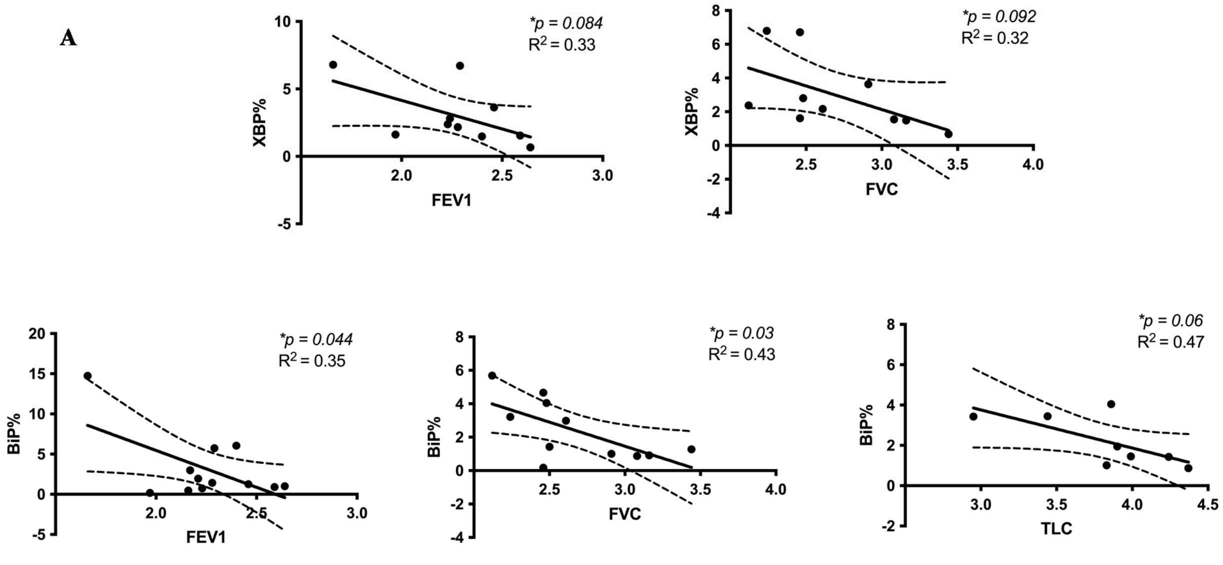

B

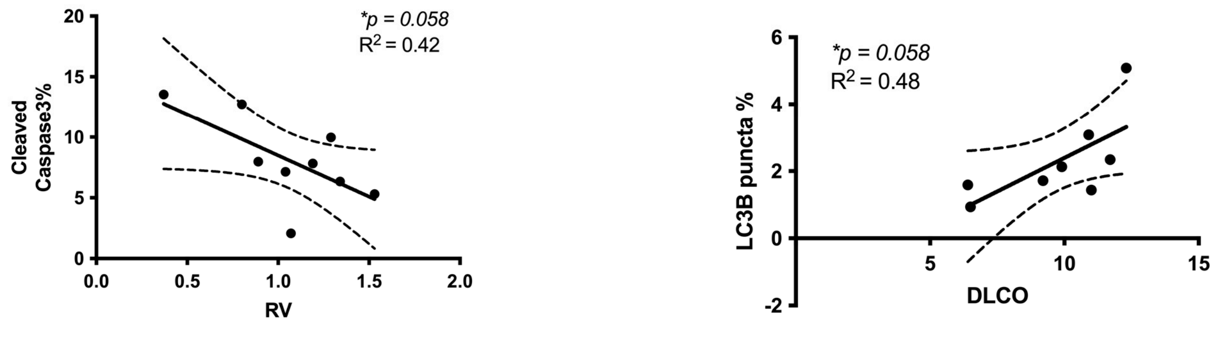

C

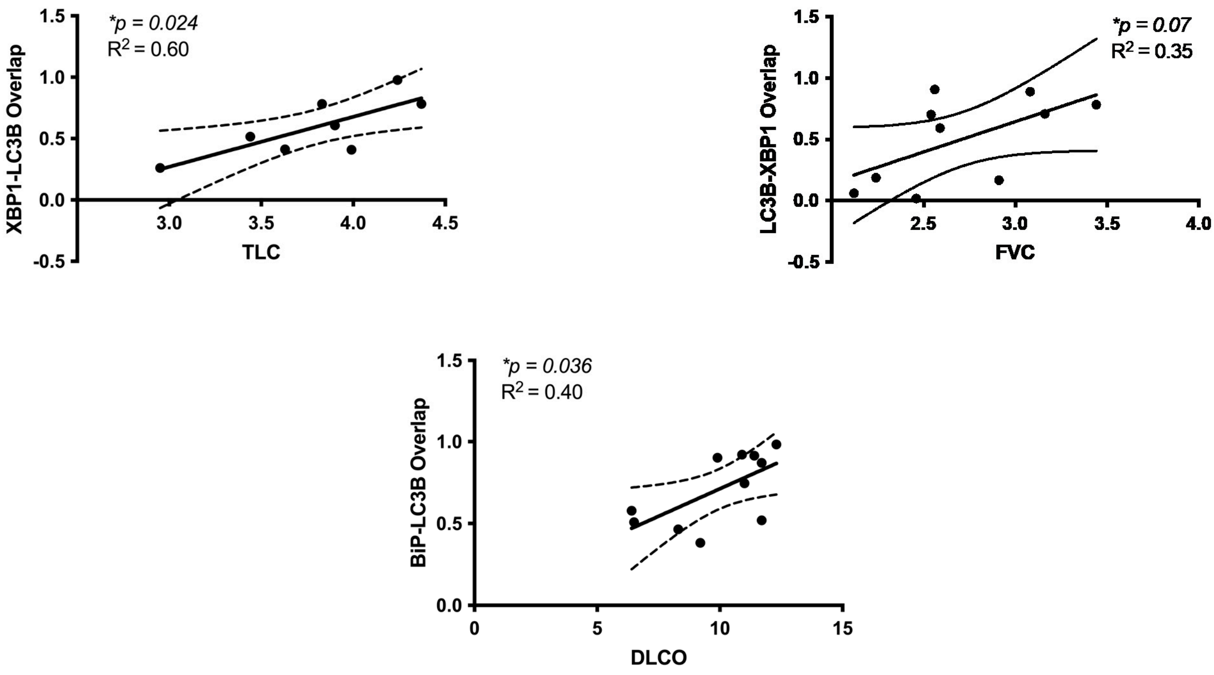

Figure S1: Correlations of Cell Stress Markers and Lung Function after Random Assignments of Data. (A,B) The UPR marker XBP1% was negatively correlated with FEV1 (*p=0.084, R^2=0.33) and FVC (*p=0.092, R^2=0.32). The UPR marker BiP% was similarly negatively correlated with FEV1 (*p=0.044, R^2=0.35), FVC (*p=0.03, R^2=0.43), and TLC (*p=0.06, R^2=0.47). The apoptosis marker cleaved caspase-3% was also negatively correlated with RV (*p=0.058, R^2=0.42). The autophagy marker LC3β puncta% was positively correlated with DLCO (*p=0.058, R^2=0.48). **(C)** Colocalized XBP1 and LC3β puncta was positively correlated with TLC (*p=0.024, R^2=0.60), colocalized LC3β puncta and XBP1 positively correlated with FVC (*p=0.07, R^2=0.35), colocalized BiP and LC3β puncta positively correlated with DLCO (*p=0.036, R^2=0.40). Confidence Intervals are included shown by the dotted curved lines flanking the regression line. Linear regression analyses were performed using GraphPad Prism 9.

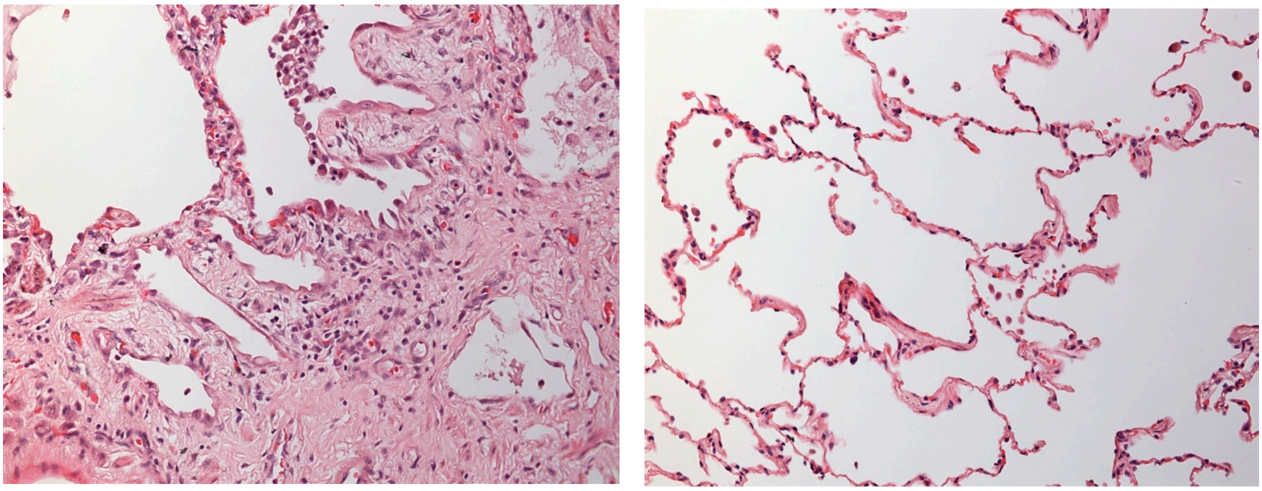

(A) **(B)**

Figure S2: Representative Lung Histopathology Images of IPF and Non-IPF Subjects. (A) H & E stained lung section from an IPF study subject showing extensive fibrotic changes in the alveolar spaces. **(B)** H & E stained lung section from a non-IPF study subject showing normal non-fibrotic alveolar spaces. Both images are at 20× magnification.
